# Valorization of Organic Third-Category Fruits Through Vinegar Fermentation: A Laboratory-Scale Evaluation of Apples, Peaches, and Clementines

**DOI:** 10.3390/foods15020238

**Published:** 2026-01-09

**Authors:** Yasmin Muhammed Refaie Muhammed, Ivana Cavoski, Carmen Aurora Apa, Giuseppe Celano, Matteo Spagnuolo, Fabio Minervini, Maria De Angelis

**Affiliations:** 1 Dipartimento di Scienze del Suolo, della Pianta e degli Alimenti, Università degli Studi di Bari Aldo Moro, 70126 Bari, Italy; yasmin.muhammed@uniba.it (Y.M.R.M.); carmen.apa@uniba.it (C.A.A.); giuseppe.celano@uniba.it (G.C.); matteo.spagnuolo@uniba.it (M.S.); maria.deangelis@uniba.it (M.D.A.); 2 CIHEAM BARI Mediterranean Agronomic Institute of Bari, 70010 Valenzano, Italy; cavoski@iamb.it

**Keywords:** organic fruits, waste-to-value strategy, fruit vinegar, apple, clementine, peach, *Saccharomyces cerevisiae*, *Gluconobacter oxydans*, acetic acid, VOCs

## Abstract

This study aimed to evaluate the feasibility of producing vinegar from organic third-category apples, peaches, and clementines on a laboratory scale. Two-step fermentation with *Saccharomyces cerevisiae* and *Gluconobacter oxydans* was applied, monitoring production of ethanol and acetic acid and microbial dynamics. Fruit vinegars were subjected to analyses of sensory traits, color, volatile organic compounds (VOCs), and antioxidant activity. Comparable ethanol yields across substrates were obtained, ensuring consistent acetous fermentation and achieving acetic acid concentrations of 5.0–5.6%. Dynamics of yeasts and acetic acid bacteria reflected the production of and subsequent decrease in ethanol. Overall, fermentation proceeded a bit faster in peach juice. Overall, the fruit vinegars, particularly those from peaches and clementines, exhibited darker and more saturated tones. The values of colorimetric indexes fell within the range reported for vinegars. Sensory analysis highlighted large differences among the vinegars. Notwithstanding the highest scores of color, aroma intensity, and floral aroma received by the peach vinegar (PV), it received the lowest acceptability. Clementine vinegar (CV) was especially appreciated. Multivariate analysis based on the VOC profile showed that apple vinegar (AV) was quite similar to the commercial one, whereas PV and CV were well distinguished from it. CV showed the highest antioxidant activity followed by PV.

## 1. Introduction

Food waste is a global challenge, with roughly one-third of all food produced—around 1.3 to 1.6 billion tons—being lost or wasted annually [1]. Fruits and vegetables are among the categories most affected by the issue of food waste, with recent FAO estimates indicating roughly 31–45% of fruit and vegetable output is lost or wasted along the supply chain [2]. Much of this waste occurs at farms and retailers due to stringent quality and aesthetic standards. So-called “second” or “third-category” fruits—those with aesthetic imperfections or irregular sizes—are often rejected despite being perfectly edible [3]. For example, in a study of apple suppliers, around 17–19% of production was culled for purely aesthetic reasons, such as blemishes or non-uniform appearance, yielding no financial benefit to growers [4]. In Europe, it is estimated that over one-third of fresh produce grown may never reach consumers because of aesthetic grading standards [5]. This practice not only represents a major economic loss for farmers and distributors but also entails significant environmental costs (greenhouse gas emissions, resource waste) associated with producing food that is ultimately never eaten. Therefore, there is a clear need for innovative strategies to re-utilize on-farm and retail fruit waste—particularly for “third-category” fruits that currently end up as waste—to improve food system sustainability.

Fermentation offers a promising pathway to valorize third-category fruits and thereby mitigate food loss. In particular, vinegar production has emerged as an attractive waste-to-value strategy in the food industry [3]. Vinegar has been produced and utilized by humans since antiquity as a preservative and condiment, and it can be made from any sugary or starchy biomass via two successive fermentations: an alcoholic fermentation by yeasts, followed by an acetic acid fermentation by acetic acid bacteria (AAB) [6,7,8]. This process converts fruit sugars into a shelf-stable product, reducing fruit waste and creating a value-added food item. Notably, the acetic fermentation imparts strong preservative and organoleptic properties to the final product, meaning that even fruits of marginal fresh-market quality can yield acceptable vinegar. Typically, vinegar classification relies on the source material used in its production [6]. Vinegar is widely produced from a range of raw materials, such as fruits, grains, and vegetables. Major categories are fruit vinegars, grain-based vinegars, alcohol-derived vinegars, and sugar or vegetable-based vinegars, produced either through traditional surface fermentation or modern submerged fermentation systems [9,10,11]. Fruits that are aesthetically unfit for sale can often be fermented into high-quality vinegars. Some case studies underscore the potential of this approach; for instance, citrus processing byproducts (like bergamot orange peels and pulp) have been converted into vinegars that are rich in bioactive compounds [12].

Vinegars produced from fruits also retain many bioactive constituents of the raw fruit, such as organic acids (e.g., acetic, malic, and citric), phenolic compounds, vitamins, and minerals, which together impart notable functional properties [13]. Studies have indicated that vinegars may have beneficial impacts on health, such as improving digestion, stimulating appetite, aiding in recovery from fatigue, reducing lipid levels, and regulating blood pressure [14,15,16,17]. A broad analysis of 23 different fruit vinegars found they contained diverse profiles of phenolics (e.g., gallic, chlorogenic, and caffeic acids) and organic acids, and exhibited considerable antioxidant capacities [18,19].

The choice of raw material used in vinegar production significantly influences the sensory and chemical characteristics of the final product [3]. Studies have been carried out on the production of vinegars from conventional fruits such as blueberry [20], strawberry [21], pomegranate [22], apple [23] and mango [24]. Apple vinegar is among the most extensively studied fruit vinegars, with research detailing acetic fermentation kinetics, organic acid composition, and profiles of phenolics and other bioactive compounds, as well as the influence of processing conditions on sensory and functional attributes [25,26]. Changes in organic acids, volatile organic compounds, antioxidant activity, and physico-chemical properties have also been reported during fermentation that yields peach vinegar [13,27]. In contrast, little work has focused on citrus-based vinegars beyond lemon or orange, and published research specifically on clementine vinegar remains scarce. This gap is noteworthy because clementines contain high levels of fermentable sugars, aromatic terpenes, and vitamin C, suggesting potential for producing vinegars with unique volatile and functional profiles.

In addition, research is needed to evaluate vinegars from organic fruits. Italy is known for its robust organic market [28], where consumers have great enthusiasm for both locally sourced organic products and those with organic certification, showing a high level of awareness regarding organic standards [29]. This growth phase is indicative of the increasing demand for organic products, which has been attributed to various factors, such as the rise in health and environmental awareness among consumers [30,31]. Organic farming has been recognized as a tool to reach the goals of sustainable food production, as well as of improving livelihoods [32]. Despite extensive research on vinegar production from conventional fruits, limited studies have investigated the valorization of organic third-category fruits, particularly peaches and clementines, through controlled fermentation processes.

Therefore, the aim of this study was to evaluate the feasibility of producing vinegar from organic third-category apple, peach, and clementine fruits at a laboratory scale. We sought to determine whether standard submerged fermentation processes (a yeast-driven alcoholic fermentation followed by AAB-driven acetic acid fermentation) can successfully convert these three fruits into vinegar, and evaluate the fermentation dynamics, volatile organic compounds (VOCs), and sensory attributes of the produced fruit-based vinegars.

## 2. Materials and Methods

### 2.1. Raw Materials and Preparation of Juices

Fresh organic apples (*Malus domestica*), peaches (*Prunus persica*), and clementines (*Citrus clementina*) were purchased from a supermarket located in Bari, Italy (NaturaSi), which were considered to have a full physiological ripeness. The fruits were characterized by full color development and an absence of microbial spoilage, but showed significant shape defects (e.g., bruised, undersized, or irregularly shaped). However, only fruits that were firm, aromatic, and free from mechanical damage were selected for vinegar production. Organic sugar and commercial unpasteurized organic apple vinegar (“Aceto di mele biologico”, Alce Nero S.p.A., Bolzano, Italy) were also purchased from the same supermarket.

**Figure 1:**
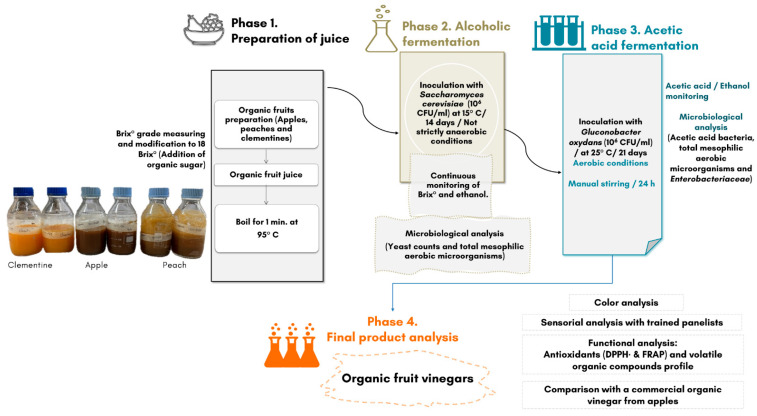
Experimental design for production of organic fruit vinegars from apples, peaches, and clementines.

Fruits were cleaned with tap water and peeled, and juices were prepared and filtered using a juice extractor (J80 Ultra, Robot-Coupe, Vincennes, France). pH and °Brix of juices were measured using pH meter (Denver Instrument, Denver, CO, USA) and Handheld refractometer (Mettler-Toledo GmbH, Greifensee, Switzerland), respectively. If needed, the juices were sweetened with organic sugar to achieve a standard concentration of 18 °Brix. Experimental plan is shown in Figure 1.

### 2.2. Microorganisms and Culture Conditions

Freeze-dried *Saccharomyces cerevisiae* DSM 1848 and *Gluconobacter oxydans* DSM 7145 (both isolated previously from beer) were purchased from Leibniz Institute DSMZ-German Collection of Microorganisms and Cell Cultures (DSMZ), Science Campus Braunschweig-Süd, Germany.

*S. cerevisiae* DSM 1848 was routinely cultured in Yeast Malt (YM) medium (yeast extract 3 g/L, malt extract 3 g/L, peptone 5 g/L, glucose 10 g/L, and pH 5.0 ± 0.1) at 20 °C for 48 h. *G. oxydans* DSM 7145 was routinely cultured in Yeast Peptone Mannitol (YPM) medium (yeast extract 5 g/L, peptone 3 g/L, and mannitol 25 g/L) at 25 °C for 48 h.

### 2.3. Fermentation of Fruit Juices to Vinegar

Juices (350 mL) from apples, clementines, and peaches were individually poured into sterilized 500 mL bottles and pasteurized at 95 °C for 1 min to reduce the native load of undesired microorganisms. After cooling to 20 °C, the juices were inoculated with *S. cerevisiae* at an initial cell density of 6 log_10_ CFU/mL. All fermentations were performed in triplicate.

The alcoholic fermentation was conducted at 15 °C for 14 days, a duration selected based on preliminary observations of ethanol stabilization typically occurring within this period in fruit-based fermentations. This ensured that an ethanol concentration of approximately 5% (*v*/*v*) was achieved, providing a sufficient substrate for the subsequent acetic fermentation while accounting for potential ethanol losses over time. Sugar and ethanol concentrations were monitored immediately after inoculation and then every 24 h. Additionally, samples were collected daily to determine yeast cell density and total mesophilic aerobic microorganisms.

For the secondary (acetous) fermentation, *G. oxydans* was added to the fermented juice at an initial cell density of 6 log_10_ CFU/mL. The acetous fermentation was carried out at 25 °C for 21 days. During this stage, ethanol and acetic acid concentrations were measured every 24 h, and the cell densities of AAB and total mesophilic aerobic microorganisms were determined daily as described below.

### 2.4. Determination of Ethanol and Acetic Acid

Ethanol concentration in the fermenting juices was estimated through the Ethanol Assay Kit (K-ETOH, Megazyme Ltd., Wicklow, Ireland), according to the manufacturer’s instructions. Carbon dioxide was removed from juices through the addition of 2 M NaOH until the pH was adjusted to approximately 9. Juices were kept at room temperature for 30 min, and then pH was measured again and corrected, if necessary. Afterwards, samples were diluted at a ratio of 1:50 (*v*/*v*) using distilled water, and they were used in the enzyme-based assay. Acetic acid concentration in the juices was determined using the Acetic Acid Assay Kit (K-ACET, Megazyme Ltd.), following the manufacturer’s instructions. Prior to analysis, samples were diluted with distilled water as necessary to ensure that acetic acid concentrations fell within the assay’s linear detection range. The absorbance of the reaction mixtures prepared for both enzyme-based assays was measured at 340 nm using an Ultrospec 3000 (Pharmacia Biotech, Uppsala, Sweden) spectrophotometer. Concentrations of ethanol and acetic acid were calculated using Megazyme Mega-Calc™ (www.megazyme.com (accessed on 4 January 2026)) and expressed as % (*v*/*v*) for ethanol and % (*w*/*v*) for acetic acid.

### 2.5. Microbiological Analyses

Presumptive yeasts and AAB, as well as total mesophilic aerobic microorganisms (TMA) in fruit juices, during and at the end of fermentation, were enumerated through plate counts. Before plating, samples were serially diluted with saline solution (NaCl 9 g/L), and 1 or 0.1 mL aliquots, depending on the inoculation technique, were inoculated in Sabouraud Dextrose Agar (SDA) for yeasts, Glucose-Yeast Extract-Calcium Carbonate (GYC) agar (50 g/L glucose, 10 g/L yeast extract, 5 g/L calcium carbonate, and 20 g/L agar) for AAB, and Plate Count Agar for TMA. SDA and Plate Count Agar were inoculated through the pour-plate technique, whereas GYC was inoculated through the spread plate technique. Plates were incubated at 30 °C for 48 h (yeasts and TMA) or for 72 h (AAB).

Additionally, total coliforms were enumerated at the beginning and end of acetous fermentation to ensure the microbiological safety of the vinegar samples for consumption. Fermented juices and vinegars were pour-plated (as such and diluted) on Violet Red Bile Glucose Agar (VRBGA). Plates were examined after incubation at 37 °C for 24 h. SDA, Plate Count Agar, and VRBGA were purchased from Oxoid, whereas GYC was prepared at laboratory.

### 2.6. Color Analysis

Instrumental color measurements of vinegar (experimental vinegars, as well as the commercial vinegar) were taken using a Konica Minolta CR-10 reader (Chiyoda, Tokyo, Japan), as described by Limongelli et al. [33]. The calibration of the instrument was performed by placing the tip of the color reader against a white blank paper. The analysis was performed according to the CIELab standard, namely using the L*, a*, and b* color space analysis approach, where L* stands for lightness (white to black) and a* (red to green) and b* (yellow to blue) for chromaticity coordinates.

### 2.7. Volatile Organic Compound Profiling

Experimental vinegars, namely juices from apples (AV), clementines (CV), and peaches (PV) at the end of acetous fermentation, were subjected to analysis of volatile organic compounds (VOCs). The commercial vinegar was used as a reference. VOCs were profiled according to the method described by Hu et al. [34], with modifications reported by Limongelli et al. [33]. Briefly, 5 mL of vinegar, 0.5 g of NaCl, and 10 μL of internal standard solution (4-methyl-2-pentanol, final concentration 33 mg/L) were added to 20 mL glass vials, sealed with magnetic screw caps equipped with PTFE/silicone septa. VOCs were extracted through headspace solid-phase microextraction (HS-SPME) using a PAL autosampler system (CTC Analytics, Zwingen, Switzerland) equipped with a 50/30 μm divinylbenzene/carboxen/polydimethylsiloxane (DVB/CAR/PDMS) fiber (Supelco, Bellefonte, PA, USA). Samples were equilibrated at 45 °C for 15 min, after which the fiber was exposed to the vial headspace at 45 °C for 45 min under continuous stirring. The extracted VOCs were thermally desorbed in the injection port of an Agilent 8890 GC system (Agilent Technologies, Santa Clara, CA, USA) operated in split-less mode at 250 °C for 3 min. Helium was used as the carrier gas at a constant flow rate of 1 mL/min. The chromatographic separation was achieved using a DB-WAX capillary column (30 m × 0.25 mm × 0.25 µm; Agilent Technologies, Santa Clara, CA, USA) under the following oven temperature program: initial temperature 35 °C (8 min), increased to 60 °C at 4 °C/min, then to 160 °C at 6 °C/min, and, finally, to 200 °C at 20 °C/min, and held for 15 min. Detection was performed with an Agilent 5977C GC/MSD quadrupole mass spectrometer (Agilent Technologies), with the ionization source set at 250 °C, the MS transfer line at 230 °C, and electron ionization at 70 eV. Mass spectra were recorded over an m/z range of 34–350. Chromatograms were processed using the NIST 2023 mass spectral library for compound identification. Only peaks with an area greater than 1,000,000 and a match probability ≥ 85% were accepted, and manual verification of fragment patterns was performed when necessary. The relative concentration of each VOC was expressed as μg/mL of 4-methyl-2-pentanol, calculated from the ratio between the peak area of each compound and that of the internal standard. All analyses were performed in triplicate.

### 2.8. Descriptive Sensory Analysis of Vinegar Samples

The experimental vinegar samples, as well as the commercial vinegar, were evaluated through descriptive sensory analysis by a panel of 10 trained assessors (aged 22–50) under controlled lighting and temperature conditions described by Wieczyńska and Cavoski [35], with some modifications. Each sample (20 mL) was presented in randomized order in coded, odorless, transparent plastic cups at room temperature (20 °C). Panelists assessed a total of 16 sensory attributes: visual (color intensity, clarity, and viscosity), odor (intensity, alcoholic, floral, cider, and citrus zest), taste (sweetness, acidity, bitterness, balance, and astringency), mouthfeel (persistence and quality of taste), and overall acceptability. For each attribute, an unstructured 15 cm line scale was used, anchored at “low” (0 cm) and “high” (15 cm) intensity. Panelists were instructed to cleanse their palates with water between tasting different samples. The evaluation involved marking the scale to reflect the perceived intensity of each attribute, and the distance from the left end of the scale to the mark was recorded as the score. Panelists were given a lexicon to have a reference for the meaning of each attribute (Supplementary Table S1).

This study did not require ethical approval as it involved only trained adult panelists evaluating non-hazardous food products.

### 2.9. Determination of Antioxidant Activity

In vitro antioxidant activity of the vinegar samples (experimental and commercial) was assessed through two complementary assays: the 2,2-diphenyl-1-picrylhydrazyl (DPPH∙) radical scavenging assay and the ferric reducing antioxidant power (FRAP) assay. The DPPH∙ radical scavenging activity was determined as described by Caponio et al. [36], with modifications reported by Limongelli et al. [37]. Briefly, a 0.08 mM DPPH∙ solution was prepared in ethanol. An aliquot of 50 μL of each sample was added to 950 μL of the DPPH∙ solution in spectrophotometric cuvettes. After incubation in the dark for 30 min at 25 °C, the absorbance was measured at 517 nm using a spectrophotometer. A mixture containing 950 μL of DPPH∙ solution and 50 μL of 80% ethanol was used as the blank, while 50 μL of butylated hydroxytoluene (BHT) solution (0.45 g/L in 80% ethanol) served as the positive control. The free radical scavenging activity was calculated as follows:

DPPH· radical scavenging activity (%) = [(A_DPPH·_ − A_sample_)/A_DPPH·_] × 100

where A_DPPH·_ is the absorbance of the blank and A_sample_ is the absorbance after reaction of the DPPH∙ solution with the sample.

The FRAP assay was performed according to the procedure described by Son et al. [38], with modifications by Minervini et al. [39]. In brief, 200 μL of each sample, diluted (1:4) with deionized water, were mixed with 200 μL of 0.2 M sodium phosphate buffer (pH 6.6) and 200 μL of 1% (*w*/*v*) potassium ferricyanide. The mixture was incubated at 50 °C for 20 min, followed by the addition of 200 μL of 10% (*w*/*v*) trichloroacetic acid to stop the reaction. After centrifugation (8000× *g*, 10 min, 4 °C), 500 μL of the resulting supernatant was combined with 400 μL of demineralized water and 100 μL of 0.1% (*w*/*v*) ferric chloride solution. After incubation for 10 min at 25 °C, the absorbance was measured at 700 nm. Ascorbic acid (1% *w*/*v*) was used as a positive control.

### 2.10. Statistical Analysis

Analyses evaluating microbiological parameters, chemical composition (including acetic acid and ethanol concentrations), sensory attributes, color, and antioxidant activity were conducted in triplicate, and results are reported as mean ± standard deviation. Statistical analyses were performed using SPSS Statistics version 28. Data was subjected to one-way analysis of variance (ANOVA) to identify significant differences among samples. When ANOVA revealed significant effects (*p* < 0.05), means were compared using Tukey’s Honestly Significant Difference (HSD) post hoc test. Data from HS-SPME-GC/MS were analyzed through principal component analysis (PCA) to evaluate differences in VOC profiles among vinegar samples. Additionally, a heatmap with cluster analysis was generated using the “pheatmap” package in R [40].

## 3. Results

### 3.1. Alcoholic Fermentation of the Fruit Juices

**Figure 2:**
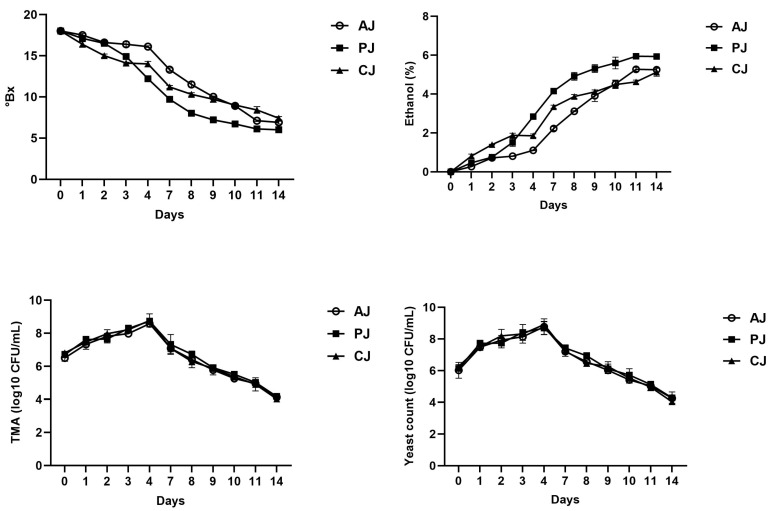
Alcoholic fermentation kinetics, expressed as °Bx, concentration of ethanol, and microbial cell densities, expressed as log_10_ CFU/mL, of fruit juices from apple (AJ), clementine (CJ), and peach (PV) during 14 days of fermentation.

Variations in °Brix, ethanol concentration, yeasts, and TMA counts were used to monitor the alcoholic fermentation of apple (AJ), peach (PJ), and clementine (CJ) juices (Figure 2). On day four, sugar concentrations had already declined from 18.0 °Brix to 16.1, 12.2, and 14.0 °Brix for AJ, PJ, and CJ, respectively. Afterwards, sugar continued to decline, and on day 14, the values decreased to 6–7.4 °Brix, with PJ showing the lowest value.

Ethanol concentration increased steadily, reaching 5.25 ± 0.11% (AJ), 5.94 ± 0.08% (PJ), and 5.12 ± 0.15% (CJ) on day 14 (Figure 2). Yeasts increased sharply during the initial fermentation phase, reaching their maximum at day four (8.7–8.9 log_10_ CFU/mL). Subsequently, they declined gradually to 4.0–4.2 log_10_ CFU/mL on day 14. TMA counts followed the same trend as yeasts (Figure 2).

### 3.2. Acetic Fermentation of the Fruit Juices

**Figure 3:**
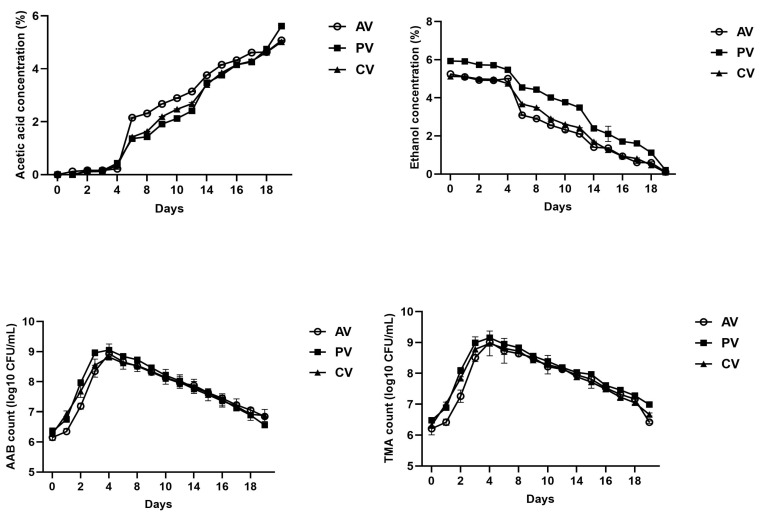
Acetic acid fermentation dynamics, expressed as concentrations of acetic acid and ethanol, and microbial cell densities, expressed as log_10_ CFU/mL, in vinegars from apple (AV), clementine (CV), and peach (PV) during 21 days of fermentation.

After inoculation with AAB, ethanol concentrations decreased progressively throughout fermentation (*p* < 0.05), confirming the oxidative activity of *G. oxydans* on that substrate (Figure 3). While in CV and AV we found that ethanol concentration overall stood quite constant during the first four days, in PV ethanol started to decrease in a quite linear trend from the first day of acetous fermentation. At the end of fermentation (day 21), ethanol reached values ranging from 0.09 (CV) to 0.21 (PV) %.

Acetic acid concentrations increased (*p* < 0.05) inversely with ethanol decline (Figure 3). A linear rise was recorded from day 3 to day 21 for PV, and, at the end of fermentation, contained 5.61 ± 0.09% of acetic acid. We found that acetic acid was almost not produced during the first four days of fermentation of CV and AV. After 21 days of fermentation, CV and AV contained acetic acid at concentrations of 5.02 ± 0.08% and 5.07 ± 0.1%, respectively (Figure 3).

After inoculation of *G. oxydans*, we found that in PV, on day three, the AAB population reached a cell density in the order of 8 log_10_ CFU/mL (Figure 3), which is in line with the more rapid decrease in ethanol and increase in acetic acid observed for this vinegar. Then, in all the vinegars, the highest values of cell density of AAB were reached on day four, ranging from 8.8 ± 0.2 log_10_ CFU/mL (CV) to 9.5 ± 0.2 log_10_ CFU/mL (PV). Afterwards, gradual declines were observed, with the final counts ranging from 6.6 ± 0.2 (PV) to 6.9 ± 0.1 (CV) log_10_ CFU/mL. TMA counts followed similar kinetics to AAB population (Figure 3). Total coliforms were below the detection limit at the beginning of fermentation and at its end (21 days) in any of the vinegars.

### 3.3. Color Indexes of the Fruit Vinegars as Compared to Commercial Vinegar

Instrumental color analysis was performed for all three experimental vinegars (CV, AV, and PV), as well as for the commercial organic apple vinegar. The latter exhibited the highest (*p* < 0.05) L* value, corresponding to the brightest appearance (Table 1). The lowest values of the L* index were found for PV and CV. The a* parameter, representing the red–green axis, significantly (*p* < 0.05) varied across vinegars. PV showed the highest redness, followed by CV and AV. For the b* parameter, which reflects the yellow–blue component, PV also exhibited the highest yellowness, followed by AV and commercial vinegar (Table 1).

### 3.4. VOC Profiles of the Fruit Vinegar as Compared to Commercial Vinegar

A total of 61 VOCs were identified across the vinegar samples (the three experimental vinegars and the commercial one), encompassing alcohols, aldehydes, carboxylic acids, esters, hydrocarbons, ketones, phenols, terpenes, and miscellaneous compounds (Supplementary Table S2). Quantitatively, AV showed a VOC profile dominated by branched-chain higher alcohols, such as 3-methyl-1-butanol (3.22 ± 1.07 µg/mL) and 2-methyl-1-butanol (2.49 ± 0.55 µg/mL), together with medium-chain fatty acids, including hexanoic (2.90 ± 0.35 µg/mL), octanoic (4.32 ± 0.31 µg/mL), decanoic (4.73 ± 0.33 µg/mL), and dodecanoic acids (1.73 ± 0.10 µg/mL). Corresponding ethyl esters, such as ethyl hexanoate (0.85 ± 0.09 µg/mL), ethyl tetradecanoate (0.29 ± 0.01 µg/mL), and ethyl hexadecanoate (0.42 ± 0.06 µg/mL), were also significantly (*p* < 0.05) higher in AV compared with the other vinegars (Supplementary Table S2). CV displayed a markedly different VOC profile, with high levels of monoterpenes and phenolic compounds, including alpha-Terpineol (4.47 ± 0.24 µg/mL), linalool (0.23 ± 0.03 µg/mL), phenylethyl alcohol (5.33 ± 2.01 µg/mL), and 2,4-bis(1,1-dimethylethyl)-phenol (4.25 ± 3.69 µg/mL). These volatiles were largely absent or present at trace levels in the other vinegars. PV exhibited an aldehyde- and acetate-ester-dominated pattern. Benzaldehyde (4.58 ± 0.30 µg/mL), furfural (1.46 ± 0.09 µg/mL), ethyl acetate (2.06 ± 0.12 µg/mL), and benzoic acid (1.56 ± 0.29 µg/mL) were among its most abundant VOCs. The commercial apple vinegar displayed the simplest VOC profile, dominated by acetic acid (5.70 ± 1.42 µg/mL). Phenylethyl alcohol (4.14 ± 2.01 µg/mL) was the only higher alcohol present at notable levels (Supplementary Table S2).

**Figure 4:**
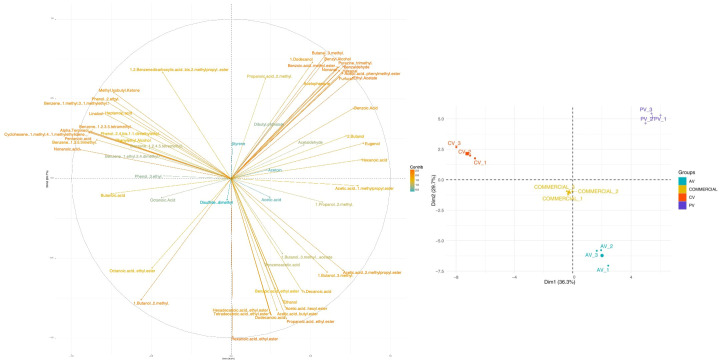
PCA of VOCs of apple vinegar (AV_1, AV_2, and AV_3), clementine vinegar (CV_1, CV_2, and CV_3), peach vinegar (PV_1, PV_2, and PV_3), and commercial apple vinegar.

Quantitative data of VOCs combined with multivariate analysis revealed clear compositional differences among the four vinegar types, as illustrated by the PCA (Figure 4).

In detail, the first two principal components of the PCA explained about 66% of the total variance. Using those two components, the vinegar types were clearly distinguished. Interestingly, CV was well-separated from the commercial vinegar, which is mainly associated with the presence of terpenic (e.g., alpha-Terpineol) and phenolic (e.g., 2,4-bis(1,1-dimethylethyl)-phenol) compounds, which are characteristic of citrus-derived matrices. PV was very well distinguished from CV because of its higher content of benzoic compounds, namely benzaldehyde and benzyl alcohol, which are typically associated with stone-fruits.

**Figure 5:**
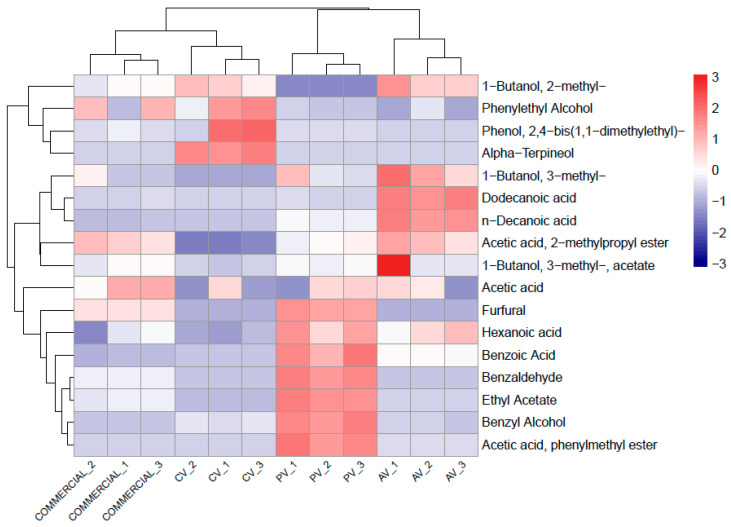
Heatmap of statistically significant VOCs (*p*-value < 0.05): VOCs in apple vinegar (AV_1, AV_2, and AV_3), clementine vinegar (CV_1, CV_2, and CV_3), peach vinegar (PV_1, PV_2, and PV_3), and commercial apple vinegar.

To better highlight the differences in the VOC profiles among the vinegars, a clustered heatmap was built using just those compounds that showed at least one statistically significant difference (Figure 5). AV samples were clustered because of their relative richness in medium-chain fatty acids, 1-butanol,2-methyl, 1-butanol,3-methyl, and acetic acid,2-methylpropyl ester. PV was characterized by high contents of hexanoic and benzoic acids, ethyl acetate, and phenylmethyl ester of acetic acid, furfural, benzaldehyde, and benzoic acid. CV samples clustered together because of their relatively high levels of alpha-Terpineol. Overall, commercial vinegar exhibited a balanced but acetic-acid-centered VOC profile (Figure 5).

### 3.5. Descriptive Sensorial Analysis

**Figure 6:**
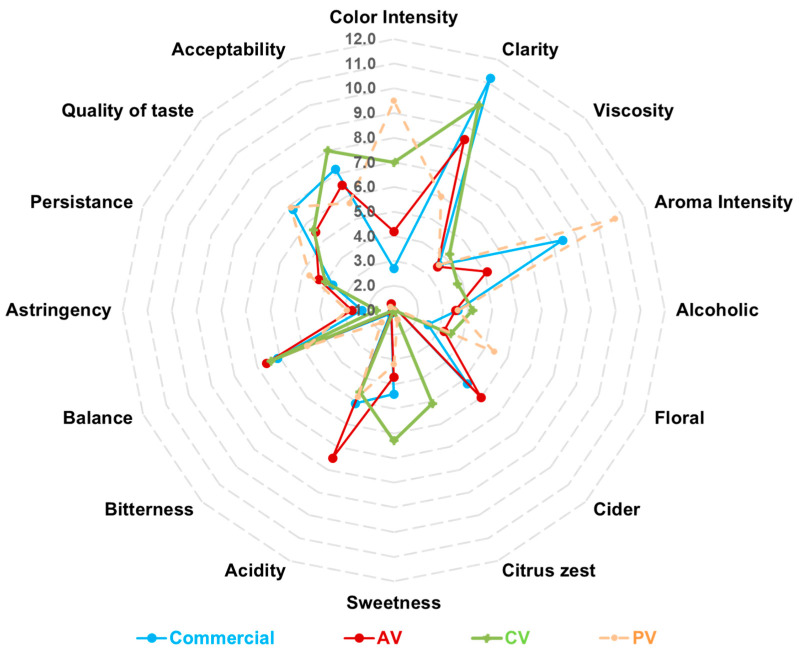
Descriptive sensory analysis of vinegars from apple (AV), clementine (CV), and peach (PV), and of commercial apple vinegar. Data are the means of scores attributed by nine trained panelists.

The descriptive sensory analysis revealed some differences among the vinegar samples (Figure 6), especially in terms of color intensity, aroma characteristics, taste attributes, and overall acceptability. PV recorded the highest color (9.5 ± 0.9) and aroma (10.7 ± 0.9) intensity, together with the strongest floral perception (5.4 ± 1.2). Despite this, PV obtained the lowest acceptability score (5.7 ± 1.4), suggesting that those intense attributes were not uniformly favored by panelists. CV was characterized by a higher sweetness perception (6.3 ± 0.8) and a notable citrus zest note (5.1 ± 0.9). It also achieved the highest acceptability rating (8.0 ± 0.7), indicating a favorable balance of taste and aroma. AV was perceived as the most acidic (7.5 ± 0.5), and its overall acceptability remained moderate (6.5 ± 0.8). Among all the vinegars, the commercial one showed comparatively lower color intensity (2.7 ± 0.4) and aroma intensity (4.9 ± 0.6) but exhibited a balanced sensory profile. It achieved high acceptability (7.2 ± 0.4) and good taste quality (6.8 ± 0.6), reflecting its role as a standard reference with consistent and familiar sensory characteristics.

### 3.6. Antioxidant Activity

**Figure 7:**
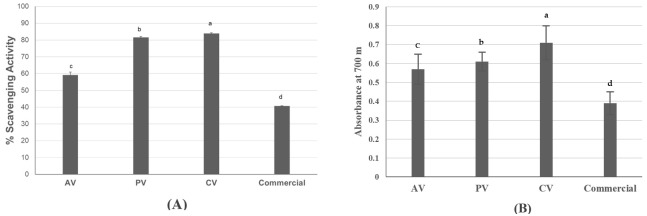
In vitro antioxidant activity of apple (AV), peach (PV), clementine (CV), and commercial vinegars, determined by DPPH· (**A**) and FRAP (**B**) assays. Different lowercase letters above the bars (a–d) indicate statistically significant (*p* < 0.05) differences.

The in vitro antioxidant capacities of the different vinegar samples were evaluated in terms of radical scavenging activity and capacity to reduce ferric to ferrous ions. CV exhibited the highest radical scavenging activity (≈84%), followed closely by PV with ≈81.5% (Figure 7A). AV showed moderate activity (≈59%), while the commercial apple vinegar displayed the lowest scavenging effect (≈41%).

Similarly, the FRAP assay (Figure 7B) revealed a consistent trend with the DPPH· results. CV showed the highest (*p* < 0.05) reducing power, with an absorbance of 0.71 U.A. ± 0.09, followed by PV (0.61 U.A. ± 0.05) and AV (0.57 U.A. ± 0.08). The commercial vinegar again showed the lowest (*p* < 0.05) ferric reducing ability (0.39 U.A. ± 0.06).

## 4. Discussion

Vinegar from fruit has attracted the attention of researchers, producers, and consumers because of its peculiar sensory traits and possible benefits to human health [3,41]. The characteristics of the raw material, microbial species involved, and fermentation conditions strongly influence process efficiency and final product quality [42]. Among those drivers, fruit characteristics, of course, play a pivotal role. In this study, we compared the susceptibility of three different fruits to be processed into vinegar, using the same microorganisms (namely *S. cerevisiae* and *G. oxydans*) and fermentation conditions, including initial concentration of sugars. All the fruits were third-category fruits from organic farming. Fruits belonging to this category are destined to be wasted with a higher probability than fruits of higher quality. We chose apples because they are the most studied for fruit vinegar, whereas clementines and peaches were considered because they are not commonly, or at all, used for vinegar production [2].

Alcoholic fermentation of all the juices proceeded rapidly during the first four days, and on day 14, they yielded an adequate ethanol concentration (5–6%) to start and complete the secondary fermentation. This result was in agreement with previous research about high-sugar fruit substrates [43,44]. Ethanol concentration at the end of the first fermentation is affected by the level of fermentable sugars in the juice and the metabolic characteristics of the yeast used [3]. We found slight differences in ethanol at the end of alcohol fermentation of the juices subjected to study, with PJ reaching the highest level. This likely reflects differences in micronutrient availability across juices, which would modulate *S. cerevisiae* metabolism and ethanol yield.

Acetic acid formation kinetics followed a similar pattern among fermented fruit juices. However, in PJ, acid accumulation began almost immediately and increased steadily, whereas AJ and CJ exhibited a lag phase of roughly four days before acid levels rose sharply. These differences highlight the influence of fruit matrix composition on AAB performance. Peach juice provided a more favorable environment for *G. oxydans*, possibly because of its higher initial ethanol concentration. Clementine’s higher levels of citric-derived components, such as limonene and linalool, along with essential oils, may have initially slowed AAB activity [45].

During alcoholic fermentation of all the juices, yeasts increased in the first days and then declined, because of ethanol accumulation and nutrients depletion, similar to strawberry and persimmon juices patterns of fermentation [46,47]. For all the fruit matrices, we found that the dynamics of yeasts and AAB reflected the production of ethanol and its subsequent decrease in favor of acetic acid. The declines in AAB population (probably represented only by *G. oxydans* used as a starter) observed after 5–6 days coincided with acetic acid concentrations surpassing 5% and the near-consumption of ethanol, which is consistent with a previous study that highlighted the dynamics of *Acetobacteraceae* in vinegar production [48]. *G. oxydans* is potentially able to oxidize several substrates, including carbohydrates and alcohols [49]. To the best of our knowledge, no study reported the use of *G. oxydans* as a pure culture for driving the fermentation of fruit juice to vinegar so far. After acetous fermentation with *G. oxydans*, all the experimental vinegars reached comparable levels of acetic acid and residual ethanol, despite putative compositional differences among fruit substrates. The experimental vinegars showed concentrations of acetic acid and ethanol in compliance with regulatory limits, namely at least 6% and lower than 1%, respectively [50]. Similar results were reported by Maske et al., who obtained apple vinegar using a natural starter containing *Saccharomyces*, *Leuconostoc*, and *Acetobacter*, among others [48]. In our study, none of the vinegars harbored coliforms at a detectable level, confirming microbiological safety, which is consistent with vinegar’s antimicrobial properties [51]. Specific testing for *Salmonella* spp. and *Escherichia coli* was not performed, as coliform counts remained below detection limits, indicating no evidence of potential fecal contamination.

Colorimetric analysis indicated that overall, the fruit vinegars produced in the laboratory, particularly those from peach and clementine juices, exhibited darker, warmer, and more saturated tones, caused by the preliminary pasteurization of the fruit juices. On the other hand, commercial vinegar was brighter but less chromatic, possibly due to industrial clarification and filtration practices. The values observed for L*, a*, and b* fell within the range reported for traditional and industrial vinegars [52]. Those color coordinates may be affected by raw material, processing conditions, and storage [53].

Sensory analysis highlighted large differences among the vinegars, assigning the highest visual intensity score to PV, followed by clementine, apple, and commercial apple vinegar; this confirmed the results of instrumental color metrics. Among other attributes evaluated by the panelists, large differences among the vinegars were found also for aroma intensity, which was quite high for PV and commercial vinegar, sweetness (the highest score attributed to CV), and acidity (the highest score attributed to AV). Interestingly, notwithstanding the highest scores of color, aroma intensity, and floral aroma received by the PV, panelists evaluated this vinegar with the lowest acceptability. It may be hypothesized that excessively intense aromatic complexity, combined with low perception of acidity, negatively affected overall acceptability. Unexpectedly, the experimental vinegar obtained from the fermentation of CJ was appreciated more than commercial vinegar, possibly due to its sweet flavor, citrus zest note, and moderate color intensity and acidity. These results would suggest that clementines are particularly suitable for producing vinegar that could be well accepted by consumers. To our knowledge, no research studies were conducted on vinegar produced from clementine, although previous studies reported the descriptive sensory analysis on experimental vinegar from other citrus fruits, such as lemon [54], mandarin [55], and orange [56]. However, they did not evaluate the overall acceptability of vinegars.

Some studies about fruit vinegar analyzed VOCs, organic acids, and free amino acids because all of them may be considered important drivers of odor, taste, and flavor of vinegar [54,57,58]. However, we preferred to focus just on VOCs because of their low odor threshold value regarding perception, compared to most organic acids and free amino acids. Profiles of VOCs for all the vinegars were consistent with the chemical diversity reported in fruit vinegars [59]. VOCs in vinegar may originate from fruits used as raw material, or may be generated by microorganisms driving the fermentation of fruit juices [60]. However, since we used the same microorganisms (*S. cerevisiae* and *G. oxydans*) for all the vinegars, we expect that most of the differences could be attributable to the different fruits used. Multivariate analysis based on VOC profile showed that our experimental AV was quite similar to the commercial apple vinegar, possibly because they were produced from juices of the same plant species. Overall, commercial vinegar showed the simplest VOC profile, possibly due to industrial processing that may have reduced volatile complexity [61]. Hexanoic acid ethyl ester, with a fruity aroma, characterized our AV, in agreement with the literature [62]. Contrary to AV, PV and CV were well distinguished from the commercial vinegar. In particular, CV was characterized by relatively high levels of alpha-Terpineol and 2,4-bis(1,1-dimethylethyl)-phenol. Alpha-Terpineol, along with linalool, probably contributed to the citrus and floral aromatic characteristics of CV, perceived by the panelists as “citrus zest note”. In addition, CV was characterized by high levels of phenylethyl alcohol, another VOC that was previously found to be typical of citrus vinegar and possibly produced by yeasts or AAB [55]. PV was characterized by VOCs, such as benzaldehyde, benzyl alcohol, benzoic acid, associated with an almond-like aroma, and ethyl acetate, associated with a fruity aroma. Budak et al. (2021) [27] showed that ethyl acetate and, especially, benzaldehyde and benzyl alcohol, increased when peach juice was processed to vinegar, whereas furfural decreased. This would suggest that yeasts or AAB are mainly involved in the dynamics of those VOCs.

In vitro antioxidant activity, determined through two assays, was highest in CV. Several compounds may confer antioxidant activity to fruit-based beverages. Anyway, previous studies highlighted strong correlations between some VOCs, such as terpenes and phenols, and antioxidant capacity [59,63]. Therefore, we hypothesize that VOCs such as alpha-Terpineol and 2,4-bis(1,1-dimethylethyl)-phenol could be among the main contributors to antioxidant activity found for CV. In addition, clementines are naturally rich in vitamin C, a potent antioxidant that could have contributed to the antioxidant activity observed for CV [64,65,66]. PV had the second most potent in vitro antioxidant activity. It had been previously shown that the antioxidant activity of peach juice could decrease or increase in the resulting vinegar, depending on the step of fermentation (alcoholic/acetous) and assay used [27]. Overall antioxidant activity of PV could be attributed to compounds such as phenols and carotenoids contained in the raw matter [67,68]. The analysis of the profile of phenolic compounds could have been helpful to try correlating the antioxidant activity of experimental vinegars with those bioactive compounds.

From a practical standpoint, the findings of this study provide important insights for potential scale-up and commercialization. Scaling up alcoholic and acetous fermentations would require careful control of oxygen supply and microbial stability, since both *S. cerevisiae* and *G. oxydans* exhibit process-sensitive kinetics that may differ in larger bioreactors. For small producers or artisanal processors, the approach tested here appears economically viable, as third-category fruits are low-cost raw materials and fermentation infrastructure can be kept relatively simple. In particular, CV presents notable commercial promise, given its high acceptability score and strong antioxidant activity, suggesting a differentiated premium product for niche markets such as gourmet, potentially functional, or locally sourced food products. The vinegars produced also showed microbiological stability and acetic acid levels compatible with long shelf life, although long-term storage behavior (clarity, sediment formation, color stability, and VOC retention) should be characterized in future work, and storage under cool and dark conditions would be advisable to preserve color and volatile profile. When compared with typical industrial vinegar production, which uses high-efficiency submerged fermentation systems capable of achieving rapid acetic acid accumulation (often within 24–48 h), the batch-type approach used here is slower but preserves more fruit-specific aroma compounds. This suggests a trade-off between production efficiency and sensory distinctiveness, with the latter offering a potential advantage for value-added artisanal products. If compared with industrial vinegar production, the process described here would be less efficient in terms of time and acetic acid yield per unit volume, but it offers added value through enhanced sensory characteristics, unique volatile profiles, and the valorization of otherwise wasted fruit resources.

## 5. Conclusions

This study demonstrated that alcoholic and acetous fermentation processes can be effectively applied to transform third-category organic fruits—specifically apples, peaches, and clementines—into vinegars with distinct chemical and sensory attributes. The resulting products reached acetic acid concentrations of 5.0–5.6% (*w*/*v*), confirming the successful conversion of low-grade fruit biomass into microbiologically stable vinegars. Among the evaluated products, CV showed particular commercial potential, supported by its high consumer acceptance score and superior antioxidant activity, suggesting strong market appeal.

Beyond their technological and sensory value, these findings underscore the potential of low-grade fruits as sustainable raw materials for producing fruit vinegars that can be appreciated by consumers. This approach could support circular bioeconomy principles by transforming underutilized or waste fruit streams into value-added products, thereby reducing environmental burden and enhancing resource efficiency. The valorization of such raw materials shows how controlled biotechnological processes can contribute to more sustainable and resilient food systems.

The methodology used in this study could be readily adapted to other fruit matrices, while future research should explore the feasibility of scaling up fermentation processes. Additionally, expanded consumer studies and further investigations into the biological activities and functional properties of fruit vinegars are recommended to strengthen the commercial and nutritional relevance of these products.

## Figures and Tables

**Table 1:** Values of color indexes found for apple (AV), clementine (CV), peach (PV), and commercial apple vinegars. Values are expressed as mean ± standard deviation (*n* = 3). Different superscript letters within the same column indicate significant differences (*p* < 0.05).

Vinegar Type	L*	a*	b*
AV	55.49 ± 0.25 ^b^	2.94 ± 0.54 ^c^	24.65 ± 0.48 ^b^
CV	54.17 ± 0.34 ^c^	4.33 ± 0.28 ^b^	19.85 ± 0.53 ^d^
PV	53.15 ± 0.53 ^c^	6.73 ± 0.36 ^a^	26.16 ± 0.22 ^a^
Commercial	58.46 ± 0.65 ^a^	1.83 ± 0.71 ^d^	21.6 ± 0.16 ^c^

## Data Availability

The original contributions presented in this study are included in the article/Supplementary Material. Further inquiries can be directed to the corresponding author.

## Supplementary Materials

The following supporting information can be downloaded at https://www.mdpi.com/article/10.3390/foods15020238/s1; Table S1: Lexicon developed for describing the sensorial attributes of the vinegar samples during the descriptive analysis; Table S2: Concentrations (in μg/mL of 4-methyl-2-pentanol) of volatile organic compounds (VOCs), in clementine (CV), apple (AV), peach (PV), and commercial apple vinegars.

## Abbreviations

The following abbreviations are used in this manuscript:

AAB	Acetic acid bacteria
TMA	Total mesophilic aerobic bacteria
PJ	Peach juice
CJ	Clementine juice
AJ	Apple juice
PV	Peach vinegar
CV	Clementine vinegar
AV	Apple vinegar
VOCs	Volatile organic compounds
DPPH∙	2,2-diphenyl-1-picrylhydrazyl
FRAP	Ferric reducing antioxidant power
