# Supplementary material for: Valorization of Organic Third-Category Fruits Through Vinegar Fermentation: A Laboratory-Scale Evaluation of Apples, Peaches, and Clementines
Source: Foods. 2026 Jan 9;15(2):238. doi: 10.3390/foods15020238 (PMC12840212; doi:10.3390/foods15020238)
Supplement: Supplementary file 1 [file foods-15-00238-s001.zip › foods-4029709-supplementary.pdf]

Supplementary Table S1. Lexicon developed for describing the sensorial attributes of the vinegar samples during the descriptive analysis

Aspect	Description
**1.Visual Appearance**	
**a. Color Intensity**	Varies from light to dark color.
**b. Clarity**	From clear to extremely cloudy.
**c. Viscosity**	Thickness of the vinegar, from light to viscous.
**2. Aroma**	
**a. Intensity**	Strength of the smell, from moderate to very intense.
**b. Alcoholic**	Alcohol smell, from absent to extremely alcoholic.
**c. Floral**	Related to peach, from slightly perceptible to extremely floral.
**d. Cider**	Related to apple, with a light acetone smell, from low to high intensity.
**e. Citrus Zest**	Spice linked to clementine, from absent to very intense.
**3. Taste and Mouthfeel**	
**a. Sweetness**	From low to high.
**b. Acidity**	From absent to extremely acidic.
**c. Bitterness**	From not bitter to extremely bitter.
**d. Balance**	Balance of ingredient flavors on the tongue, from absent to extremely balanced.
**e. Astringency**	From mild to moderate.
**4. Aftertaste**	
**a. Persistence**	Duration and intensity of the sensory impression, from short to long.
**b. Final Quality**	Evaluation of the impression left in the mouth after swallowing the vinegar.

Supplementary Table S2. Concentrations (in μg/mL of 4-methyl-2-pentanol) of Volatile Organic Compounds (VOCs), in clementine (CV), apple (AV), peach (PV), and commercial apple vinegars.

Class	Compound	AV	CV	PV	Commercial
Alcohols (10)	Benzyl Alcohol	0.052 ± 0.019[a]	0.168 ± 0.054[a]	1.214 ± 0.101[a]	0.001 ± 0.002[b]
	1-Dodecanol	0.051 ± 0.005[a]	0.075 ± 0.008[a]	0.17 ± 0.04[a]	0.103 ± 0.034[a]
	1-Butanol, 3-methyl-	3.222 ± 1.067[a]	n.d.*	1.529 ± 1.044[b]	0.912 ± 0.712[b]
	1-Butanol, 3-methyl-, acetate	3.931 ± 2.845[a]	0.526 ± 0.109[c]	1.716 ± 0.239[b]	1.831 ± 0.544[b]
	1-Butanol, 2-methyl-	2.492 ± 0.546[a]	2.131 ± 0.417[a]	n.d.	1.451 ± 0.169[b]
	Phenylethyl Alcohol	1.76 ± 0.92[c]	5.332 ± 2.011[a]	2.091 ± 0.234[c]	4.143 ± 2.012[b]
	Ethanol	0.713 ± 0.182[a]	n.d.	0.127 ± 0.01[a]	0.558 ± 0.375[a]
	1-Propanol, 2-methyl-	0.341 ± 0.047[a]	0.198 ± 0.032[a]	0.321 ± 0.076[a]	0.169 ± 0.083[a]
	2-Butanol	0.006 ± 0.009[a]	n.d.	0.015 ± 0.002[a]	n.d.
	Alpha-Terpineol	0.128 ± 0.009[b]	4.474 ± 0.24[a]	0.134 ± 0.022[b]	0.042 ± 0.038[b]
Aldehydes (6)	Benzaldehyde	0.03 ± 0.002[b]	0.032 ± 0.004[b]	4.58 ± 0.299[a]	1.029 ± 0.104[b]
	Nonanal	0.161 ± 0.032[a]	0.154 ± 0.019[a]	0.59 ± 0.011[a]	0.15 ± 0.022[a]
	Butanal, 3-methyl-	n.d.	0.009 ± 0.002[a]	0.069 ± 0.011[a]	0.006 ± 0.006[a]
	Hexanal	0.005 ± 0.001[a]	n.d.	0.109 ± 0.013[a]	n.d.
	Acetaldehyde	n.d.	n.d.	0.001 ± 0.001[a]	0.001 ± 0.002[a]
	Furfural	n.d.	n.d.	1.461 ± 0.092[a]	0.821 ± 0.017[a]
Carboxylic Acids (18)	Benzeneacetic acid	0.695 ± 0.153[a]	0.355 ± 0.069[a]	0.359 ± 0.095[a]	0.154 ± 0.014[a]
	Butanoic acid	0.218 ± 0.021[a]	0.362 ± 0.028[a]	0.087 ± 0.009[a]	0.055 ± 0.006[a]
	Pentanoic acid	0.018 ± 0.009[a]	0.328 ± 0.016[a]	0.016 ± 0.001[a]	0.009 ± 0.002[a]
	Acetic acid, 2-methylpropyl ester	2.362 ± 0.329[b]	0.541 ± 0.03[a]	1.713 ± 0.158[b]	2.225 ± 0.22[b]
	Heptanoic acid	0.038 ± 0.004[a]	0.123 ± 0.013[a]	0.052 ± 0.009[a]	0.013 ± 0.012[a]
	Nonanoic acid	0.126 ± 0.023[a]	0.367 ± 0.025[a]	0.097 ± 0.029[a]	0.159 ± 0.036[a]
	Octanoic Acid	4.319 ± 0.306[a]	4.604 ± 0.301[a]	3.976 ± 0.502[a]	4.217 ± 0.795[a]
	Ethyl Acetate	0.228 ± 0.016[b]	0.016 ± 0.027[b]	2.056 ± 0.119[a]	0.489 ± 0.055[b]
	Hexanoic acid	2.901 ± 0.348[a]	1.811 ± 0.123[b]	3.421 ± 0.396[a]	2.119 ± 0.545[b]
	Dodecanoic acid	1.728 ± 0.101[a]	0.114 ± 0.016[b]	0.119 ± 0.047[b]	0.145 ± 0.025[b]
	n-Decanoic acid	4.728 ± 0.33[a]	0.488 ± 0.006[b]	1.573 ± 0.206[b]	0.447 ± 0.063[b]
	Acetic acid	3.461 ± 2.346[b]	2.299 ± 1.448[c]	3.816 ± 2.529[b]	5.698 ± 1.42[a]
	Benzoic Acid	0.604 ± 0.037[a]	0.145 ± 0.02[b]	1.557 ± 0.288[a]	0.057 ± 0.02[b]
	Propanoic acid, 2-methyl-	0.994 ± 0.107[a]	1.18 ± 0.033[a]	1.389 ± 0.059[a]	1.424 ± 0.083[a]
	1,2-Benzenedicarboxylic acid, bis(2-methylpropyl) ester	0.329 ± 0.104[a]	0.594 ± 0.053[a]	0.493 ± 0.046[a]	0.315 ± 0.05[a]
	Dibutyl phthalate	0.313 ± 0.172[a]	0.307 ± 0.049[a]	0.393 ± 0.046[a]	0.245 ± 0.064[a]

	Benzoic acid, methyl ester	0.004 ± 0.003[a]	0.005 ± 0.004[a]	0.188 ± 0.016[a]	n.d.
	Eugenol	0.5 ± 0.035[a]	0.013 ± 0.003[a]	0.863 ± 0.143[a]	0.01 ± 0.003[a]
Carboxylic Ester (9)	Propanoic acid, ethyl ester	0.079 ± 0.008[a]	n.d.	0.009 ± 0.001[a]	0.018 ± 0.007[a]
	Octanoic acid, ethyl ester	0.168 ± 0.019[a]	0.166 ± 0.013[a]	0.031 ± 0.01[a]	0.207 ± 0.106[a]
	Hexanoic acid, ethyl ester	0.849 ± 0.086[a]	0.253 ± 0.006[a]	0.049 ± 0.004[a]	0.456 ± 0.02[a]
	Acetic acid, butyl ester	0.053 ± 0.008[a]	n.d.	0.003 ± 0.001[a]	n.d.
	Tetradecanoic acid, ethyl ester	0.29 ± 0.013[a]	n.d.	n.d.	n.d.
	Acetic acid, phenylmethyl ester	0.141 ± 0.015[b]	0.041 ± 0.003[c]	1.85 ± 0.246[a]	0.034 ± 0.003[c]
	Acetic acid, hexyl ester	0.385 ± 0.039[a]	n.d.	0.044 ± 0.004[a]	0.009 ± 0.008[a]
	Hexadecanoic acid, ethyl ester	0.419 ± 0.062[a]	n.d.	n.d.	n.d.
	Benzoic acid, ethyl ester	0.284 ± 0.024[a]	0.063 ± 0.005[a]	0.078 ± 0.008[a]	0.004 ± 0.002[a]
Hydrocarbons (6)	Styrene	0.027 ± 0.01[a]	0.031 ± 0.007[a]	0.03 ± 0.006[a]	n.d.
	Benzene, 1,3,5-trimethyl-	n.d.	0.009 ± 0.003[a]	n.d.	n.d.
	Benzene, 1,2,3,5-tetramethyl-	n.d.	0.005 ± 0.002[a]	n.d.	n.d.
	Phenol, 3-ethyl-	0.073 ± 0.008[a]	0.261 ± 0.451[a]	n.d.	n.d.
	Benzene, 1-ethyl-2,4-dimethyl-	n.d.	0.013 ± 0.023[a]	n.d.	n.d.
	Benzene, 1,2,4,5-tetramethyl-	n.d.	0.001 ± 0.002[a]	n.d.	n.d.
Ketones (3)	Methyl Isobutyl Ketone	0.029 ± 0.011[a]	0.108 ± 0.003[a]	0.056 ± 0.012[a]	0.048 ± 0.017[a]
	Acetoin	2.193 ± 0.578[a]	1.976 ± 0.371[a]	2.521 ± 0.116[a]	2.565 ± 1.506[a]
	Acetophenone	0.022 ± 0.003[a]	0.02 ± 0.001[a]	0.054 ± 0.003[a]	0.006 ± 0.003[a]
Phenol (2)	Phenol, 2-ethyl-	0.066 ± 0.006[a]	0.79 ± 0.075[a]	0.247 ± 0.033[a]	0.009 ± 0.004[a]
	Phenol, 2,4-bis(1,1-dimethylethyl)-	0.017 ± 0.013[b]	4.254 ± 3.686[a]	0.312 ± 0.049[b]	0.455 ± 0.353[b]
Terpenes (3)	Benzene, 1-methyl-3-(1-methylethyl)-	0.001 ± 0.001[a]	0.043 ± 0.003[a]	0.011 ± 0.002[a]	0.001 ± 0.001[a]
	Cyclohexene, 1-methyl-4-(1-methylethylidene)-	n.d.	0.034 ± 0.002[a]	n.d.	n.d.
	Linalool	0.011 ± 0.006[a]	0.227 ± 0.032[a]	0.043 ± 0.013[a]	n.d.
Others (3)	Acetic acid, 1-methylpropyl ester	0.061 ± 0.021[a]	0.001 ± 0.002[a]	0.07 ± 0.015[a]	0.001 ± 0.002[a]
	Pyrazine, trimethyl-	n.d.	n.d.	0.027 ± 0.002[a]	n.d.
	Disulfide, dimethyl	n.d.	n.d.	n.d.	0.002 ± 0.001[a]

[a-d] Within the same row, mean values ± standard deviation (n = 3) with different superscript letters indicate significant differences ($p < 0.05$), two-way ANOVA with Tukey's post hoc test.

[*] n.d., not detected
